# Melatonin Ameliorates Organellar Calcium Homeostasis, Improving Endoplasmic Reticulum Stress-Mediated Apoptosis in the Vastus Lateralis Muscle of Both Sexes of Obese Diabetic Rats

**DOI:** 10.3390/antiox14010016

**Published:** 2024-12-26

**Authors:** Diego Salagre, Miguel Navarro-Alarcón, Luis Gerardo González, Mohamed A. Elrayess, Marina Villalón-Mir, Rocío Haro-López, Ahmad Agil

**Affiliations:** 1 Department of Pharmacology, School of Medicine, University of Granada, 18016 Granada, Spain; dsalagre@ugr.es (D.S.); lggonzal@ugr.es (L.G.G.); rociohl@correo.ugr.es (R.H.-L.); 2 Nutrition, Metabolism, Growth and Development Group, BioHealth Institute Granada (ibs.GRANADA), 18012 Granada, Spain; 3 Neuroscience Institute “Federico Olóriz”, Biomedical Research Center (CIBM), University of Granada, 18016 Granada, Spain; 4 Department of Nutrition and Bromatology, School of Pharmacy, University of Granada, 18071 Granada, Spain; nalarcon@ugr.es (M.N.-A.); marinavi@ugr.es (M.V.-M.); 5 Bola de Oro Primary Care Health Center, Sanitary District of Granada, Andalusian Health Services (SAS), 18008 Granada, Spain; 6 Biomedical Research Center, College of Medicine, QU Health, Qatar University, Doha P.O. Box 2713, Qatar; m.elrayess@qu.edu.qa

**Keywords:** melatonin, obesity, type 2 diabetes, skeletal muscle, organellar calcium, endoplasmic reticulum stress, apoptosis

## Abstract

Endoplasmic reticulum (ER) stress is a crucial factor in the progression of obesity-related type 2 diabetes (diabesity), contributing to skeletal muscle (SKM) dysfunction, calcium imbalance, metabolic inflexibility, and muscle atrophy. The ER and mitochondria together regulate intracellular calcium levels, and melatonin, a natural compound with antioxidant properties, may alleviate these challenges. Our previous research showed that melatonin raises intracellular calcium and preserves muscle structure by enhancing mitochondrial function in obese diabetic rats. This study further explores melatonin’s potential to reduce ER stress in the vastus lateralis (VL) muscle by modulating the unfolded protein response (UPR) and restoring calcium levels disrupted by diabesity. Five-week-old Zücker diabetic fatty (ZDF) rats and lean littermates of both sexes were divided into control and melatonin-treated groups (10 mg/kg/day for 12 weeks). Flame atomic absorption spectrometry results showed that melatonin restored VL intraorganellar calcium homeostasis, increasing calcium levels in mitochondria and reducing them in the ER by raising the activity and expression of calcium transporters in both sexes of ZDF rats. Melatonin also decreased ER stress markers (GRP78, ATF6, IRE1α, and PERK) and reduced pro-apoptosis markers (Bax, Bak, P-JNK, cleaved caspase 3 and 9) while increasing Bcl2 levels and melatonin receptor 2 (MT2) expression. These findings suggest that melatonin may protect against muscle atrophy in obese and diabetic conditions by mitigating ER stress and calcium imbalance, highlighting its therapeutic potential.

## 1. Introduction

Obesity and its related type 2 diabetes mellitus (T2DM) present a global health challenge and have a significant impact on skeletal muscle (SKM) health by affecting SKM mass, structure, metabolism, and function [1,2], mainly by inducing organellar stress and dysfunction, particularly affecting the endoplasmic reticulum (ER). SKM organellar stress has recently gained importance as a crucial factor in the pathogenesis of obesity and its associated T2DM (diabesity) [3].

The ER, a pivotal organelle within the cell, is crucial for lipid, steroid, and protein synthesis; protein folding and modification; maintaining protein homeostasis; and calcium handling [4]. Mitochondria and ER are key organelles in intracellular calcium homeostasis, more precisely, regulating intraorganellar calcium levels [5]. The most studied organellar calcium transporters are the Ca^2+^-ATPase channels, the sarco/endoplasmic reticulum Ca^2+^-ATPase (SERCA), and the mitochondrial Ca^2+^-ATPase, which transports calcium to the organellar lumen. Furthermore, the ryanodine receptor (RyR) plays a pivotal role in ER calcium homeostasis, releasing calcium from intracellular stores to the cytosol [6]. The mitochondrial calcium uniporter (MCU) internalizes calcium from the cytosol into the mitochondria, also regulating intramitochondrial calcium levels [7]. Therefore, pathophysiological states that disrupt calcium transporter function promote organellar stress and cellular dysfunction. ER stress causes an accumulation of misfolded proteins that overload the functions of this organelle. At the onset of ER stress, the unfolded protein response (UPR), an intricate signaling system designed to reestablish cellular homeostasis, is activated via three primary signaling branches: the inositol-requiring enzyme 1A (IRE1α), the activating transcription factor 6 (ATF6), and the protein kinase RNA-like endoplasmic reticulum kinase (PERK) pathways [8]. When unfolded proteins accumulate in the ER, the ER chaperone protein glucose-regulated protein 78 kDa (GRP78-BiP) is dissociated, leading to the activation of the UPR cascade through the dimerization and autophosphorylation of PERK (P-PERK) and IRE1α (P-IRE1α). The regulated intramembrane is translocated to the Golgi and proteolysis of ATF6, which is then internalized in the nucleus and acts as a transcription factor. Following PERK activation, the eukaryotic translation initiation factor 2A (eIF2α) is phosphorylated (P-eIF2α), inhibiting global translation and protein synthesis. Downstream activation of activating transcription factor 4 (ATF4) promotes the expression of ER-restoring genes and synthesis of protein chaperones, particularly GRP78. While these pathways aim to alleviate ER stress, chronic or excessive activation of UPR can initiate apoptotic pathways to eliminate damaged cells when adaptive responses fail to restore cellular homeostasis [8]. ER stress-induced apoptosis is activated by the phosphorylation of the c-Jun amino-terminal kinase (P-JNK), leading to the suppression of B cell lymphoma 2 (Bcl2) and the induction of Bcl2-associated X protein (Bax) and Bcl2 homologous antagonist killer (Bak), forming the pro-apoptotic complex that binds to the outer mitochondrial membrane, subsequently activating the caspase family cleavage, mainly caspase 9 and 3 [9]. This is particularly relevant in skeletal muscle, where ER stress has been implicated in SKM cell death, muscle atrophy, metabolic inflexibility, and decreased SKM mass and functionality [10], which are hallmarks of diabesity.

Melatonin is a natural indoleamine synthesized primarily at night in the pineal gland and other tissues, which is also present in many plant-derived dietary foods and spices [11]. Apart from its well-known role in regulating circadian rhythms, a broad spectrum of biological activities of melatonin are described, including SKM cell-protective capacity [12] due to its potent cellular antioxidant, and anti-inflammatory and anti-apoptotic defense-inducing properties [13,14], which are critical in mitigating oxidative stress. Apoptosis and autophagy are key processes in cellular homeostasis in which ER and mitochondria play a fundamental role, both organelles being targets of melatonin in cell survival [14,15]. Our group has shown that melatonin restores ER-induced apoptosis in the kidney of obese diabetic rats via IRE1α [16]. Moreover, several studies in obese rodents and humans found melatonin to reduce adiposity, showing its role in energy metabolism regulation [17] and SKM health [12]. Recently, we have shown that melatonin treatment, in the same strain of obese diabetic rats, plays a key role in SKM calcium homeostasis [18]. It also reduces mitochondrial oxidative stress and nitrite levels, increasing the Mn- and CuZn-superoxide dismutase (SOD) activity. Melatonin preserves the structure, mass, and oxidative function in the vastus lateralis (VL) muscle [19], the major component of the rodents’ quadriceps, in Zücker diabetic fatty (ZDF) rats, an animal model of diabesity which exhibits characteristics analogous to human type 2 diabetes and obesity, including progressive insulin resistance, glucose intolerance, hyperglycemia, hyperinsulinemia, hyperlipidemia, moderate hypertension, and progressive SKM injury [20]. Melatonin is a highly lipophilic molecule that crosses easily through cellular membranes and produces its cellular functions through diverse mechanisms. First, it interacts with specific G-protein coupled membrane receptors leading down-stream second messengers, activation of signaling pathways, and gene transcription [21,22]. Second, it acts as a free-radical-scavenger detoxifying ROS/NOS [21,23]. Third, it binds to nuclear proteins [24,25] and intracellular proteins such as calmodulin (CaM), regulating intracellular Ca^2+^ signaling pathways because CaM is an essential mediator of Ca^2+^ signaling within cells [26,27,28]. Furthermore, melatonin, through membrane melatonin receptor 1 (MT1) and/or 2 (MT2) activation, is proposed to play a pivotal role in calcium signaling [29,30], modulating intracellular levels by regulating calcium transport [31], and in ER stress under diverse pathophysiological conditions [32]. As calcium signaling is crucial in ER stress-induced pro-apoptotic processes under pathological conditions [33], the present study aims to further investigate the SKM protective role of melatonin in modulating intraorganellar calcium levels and inhibiting ER stress-mediated apoptosis in the VL of both sexes of obese diabetic rats.

## 2. Materials and Methods

### 2.1. Reagents

All chemicals and reagents were of analytical grade and high purity and were purchased from Sigma-Aldrich (Madrid, Spain).

### 2.2. Ethical Statement

The study adhered to the ethical protocols established by the University of Granada, following European Union guidelines for animal care and protection. Ethical approval was granted under the code 23/06/2021/096-CEEA.

### 2.3. Animals and Experimental Protocol

Five-week-old male and female Zücker diabetic fatty (ZDF) rats (fa/fa; N = 16 males, N = 16 females) and lean littermates (ZL; fa/-; N = 16 males, N = 16 females) were obtained for the study. The rats were housed in pairs in climate-controlled plastic cages with a temperature of 28–30 °C, 30–40% humidity, and a light/dark cycle of 12 h. After a 4-day acclimatization period, the animals were randomly divided into control and melatonin-treated groups (10 mg/kg/day via drinking water) for 12 weeks. Throughout the study period, the melatonin solution was prepared fresh every two days and adjusted according to body weight (BW) and water intake (Supplementary Table S1) according to the following formula for each cage:

$$Melatonin\;(mg)=\frac{No.\;of\;rats\times Dosage\left(mg/kg/day\right)\times No.\;of\;days\times Mean\;rat\;BW\;(kg)\times Bottle\;volume\;(mL)}{Total\;water\;intake\;(mL)}$$

Water bottles were shielded with aluminum foil to prevent light degradation and ensure melatonin dosage stability in the solution [34]. Male rats were fed Purina #5008 chow, and females were provided Research Diet #D12468 chow, and both had ad libitum access to food and water [35]. ZDF rats are commonly used as a model for metabolic syndrome because they develop obesity, dyslipidemia, hyperglycemia, hyperinsulinemia, and T2DM as early as 9–10 weeks, progressing to complications around 20 weeks [20]. The ZDF model develops similar pathogenesis to human diabesity with some limitations for developing hyperglycemia per se [36] that can be supplied by the specific diet recommended by the manufacturer. At the end of the study, animals were anesthetized and euthanized with sodium thiobarbital (thiopental).

### 2.4. Mitochondrial and ER Fraction Isolation from VL

Approximately 200–400 mg of VL muscle tissue was dissected, and mitochondrial and ER fractions were isolated using a sequential centrifugation method which maintains organellar function [37]. Protein concentration was determined using the Bradford method.

### 2.5. VL Mineralization and Ca^2+^ Amount Analysis

To quantify the Ca^2+^ amount, 150–200 mg of whole VL tissue (total), mitochondrial, and ER fractions from VL muscle were weighed. These samples were digested and mineralized, and then, Ca^2+^ in this solution was measured using an inductively coupled plasma mass spectrometer (ICP-MS; Agilent 8900 Triple Quadrupole ICP-MS/MS, Agilent Technologies Inc., Santa Clara, CA, USA) equipped with a helium collision cell [18]. Ca^2+^ concentrations obtained were expressed as mg/g of fresh weighed tissue. The detection limit was determined in 12 blanks, yielding a concentration of 0.45 mg/L. A certified reference standard for Ca^2+^ (Bovine muscle powder nº 8414, National Institute for Standards and Technology, Gaithersburg, MD, USA) was used to assess the accuracy and precision of the technique.

### 2.6. Ca^2+^ Transporter Activity Measurement

Mitochondrial Ca^2+^-ATPase activity was measured spectrophotometrically by detecting phosphate ions released during ATP hydrolysis. Mitochondrial fractions were incubated with ATP and calcium, and absorbance was recorded at 850 nm [38]. The activity was expressed as nmol Pi/min/mg protein.

RyR activity was quantified in the ER fraction samples following a previously validated fluorometric method with minor variation [39,40]. VL ER fraction (0.5 mg/mL) and 0.25 μM of the calcium indicator fluorochrome BAPTA-Oregon Green 488 (cat#O6807, Thermofisher Scientific, Madrid, Spain) were added to 1 mL of Tris-buffered saline (TBS; 150 mM NaCl, 50 mM Tris-HCl, pH 7.6). The baseline fluorescence was initially measured without the addition of thapsigargin, a SERCA pump inhibitor. Subsequently, a pulse of 1 μM thapsigargin was added, and the fluorescence was recorded after 1 min using the fluorometer Infinite F200 (TECAN, Männedorf, Switzerland). The increase in fluorescence observed corresponded mainly to an increase in calcium transported into the cytosol by RyR channels. RyR activity was expressed as nmol Ca^2+^/min/mg protein from four replicates for each sample.

### 2.7. Protein Isolation and Expression Analysis by Western Blot

An RIPA lysis buffer containing 1% phosphatase and a protease inhibitor cocktail (cat#P0001 and cat#P8340 respectively, Sigma-Aldrich, Madrid, Spain) was used to extract total proteins from VL tissue. The Bradford method was performed to quantify protein concentrations in samples. For Western blotting, 30–50 µg of proteins underwent SDS-PAGE separation, and then, proteins were transferred to a nitrocellulose membrane [35]. Then, the membranes were blocked for 1.5 h at room temperature (RT) in Phosphate Buffer Saline (PBS) (137 mM NaCl, 10 mM Na_2_HPO_4_, 2.7 mM KCl and 1.8 mM KH_2_PO_4_) containing 1% Tween-20 (PBS-T) and 5% Bovine Serum Albumin (BSA). Primary antibodies (Supplementary Table S2) were incubated overnight at 4 °C in a 1:1000 dilution of PBS-T supplemented with 0.5% BSA followed by three 15 min washes in PBS-T. Then, horseradish peroxidase (HRP)-conjugated secondary antibodies (Supplementary Table S2) were incubated at RT for 2 h in a 1:2000 dilution of PBS-T supplemented with 0.5% BSA followed by three 15 min washes in PBS-T. A Clarity Western ECL commercial kit (BioRad, Madrid, Spain) and Image Station 4000MM Pro Molecular Imaging system (Kodak, Rochester, NY, USA) were used for protein detection according to the manufacturers’ protocols, and protein expression analysis was performed using Fiji 2.9.0 (ImageJ, National Institutes of Health, Stapleton, NY, USA).

### 2.8. Statistical Analysis

Data were presented as mean ± standard deviation (SD) and were obtained from experiments performed in triplicate, with each experiment repeated at least twice in each rat to ensure reliability. Comparisons between experimental groups were made using one-way analysis of variance (ANOVA) and Tukey’s post-hoc test. Statistical significances between the groups were defined as a *p*-value of less than 0.05. Data analysis was carried out using SPSS software version 28 (IBM, Armonk, NY, USA).

## 3. Results

### 3.1. Effects of Melatonin on VL Calcium Homeostasis

**Figure 1:**
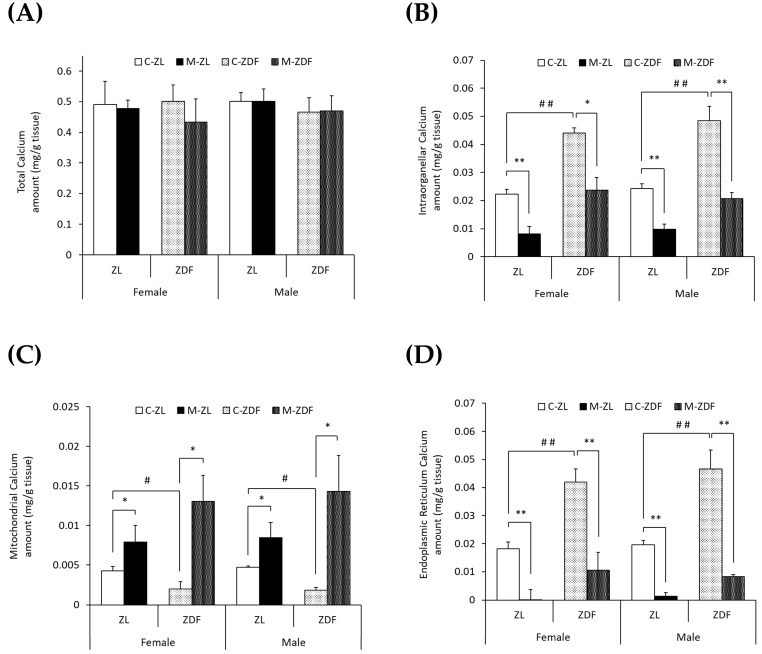
Melatonin’s effects on VL calcium levels (mg/g tissue) in female and male Zücker lean (ZL) and Zücker diabetic fatty (ZDF) rats. (**A**) Calcium levels in whole VL tissue (total). (**B**) Calcium levels in isolated VL mitochondria and endoplasmic reticulum (ER)-rich fractions (intraorganellar). (**C**) Calcium levels in isolated VL mitochondria-rich fraction. (**D**) Calcium levels in isolated VL ER-rich fraction. Results are expressed as mean ± S.E.M. of eight animals/group in triplicate. One-way ANOVA and Tukey’s post-test were performed for statistical analysis (* *p* < 0.05 and ** *p* < 0.01 melatonin vs. control rats; # *p* < 0.05 and ## *p* < 0.01 C-ZDF vs. C-ZL rats).

To study the effects of diabesity and melatonin treatment on VL calcium homeostasis in both sexes of ZDF rats, we measured the calcium levels in the whole VL muscle (total), mitochondria-enriched fractions, and ER-enriched fractions as well as the activity and expression of different calcium transporters. No significant differences were found in the VL weight of obese diabetic animals compared to lean ones or to melatonin-treated ones (Supplementary Table S1). As shown in Figure 1A, no differences in total calcium levels (mg/g tissue) were found between the two phenotypes of the same sex or between sexes. However, as shown in Figure 1B, obese diabetic rats of both sexes presented doubled intraorganellar calcium levels compared to their respective lean control rats (*p* < 0.01). After melatonin treatment, intraorganellar calcium amount was recovered in ZDF female (*p* < 0.05) and male rats (*p* < 0.01). Melatonin also reduced intraorganellar calcium levels in ZL rats of both sexes (*p* < 0.01).

Furthermore, as illustrated in Figure 1C,D, the mitochondrial calcium levels were found to be decreased, while those of the ER were found to be increased in both sexes of obese diabetic rats (female C-ZDF, 0.0020 ± 0.0009 mg/g tissue and 0.042 ± 0.005 mg/g tissue; male C-ZDF, 0.0018 ± 0.0004 mg/g tissue and 0.047 ± 0.007 mg/g tissue for mitochondrial and ER calcium levels, respectively) compared to their respective lean control rats (female C-ZL, 0.0043 ± 0.0006 mg/g tissue and 0.018 ± 0.002 mg/g tissue; *p* < 0.05; male C-ZL, 0.0047 ± 0.0004 mg/g tissue and 0.020 ± 0.002 mg/g tissue; *p* < 0.01; for mitochondrial and ER calcium levels, respectively). Melatonin treatment showed a counteracting effect in both sexes and phenotypes of rats, with intraorganellar calcium levels increasing in mitochondria (female M-ZDF, 0.0130 ± 0.0033 mg/g tissue; M-ZL, 0.0079 ± 0.0021 mg/g tissue; male M-ZDF, 0.0143 ± 0.0046 mg/g tissue; M-ZL, 0.0084 ± 0.0019 mg/g tissue; *p* < 0.05; Figure 1C) and decreasing in ER (female M-ZDF, 0.011 ± 0.006 mg/g tissue; M-ZL, 0.001 ± 0.004 mg/g tissue; male M-ZDF, 0.008 ± 0.001 mg/g tissue; M-ZL, 0.001 ± 0.001 mg/g tissue; *p* < 0.01; Figure 1D).

**Figure 2:**
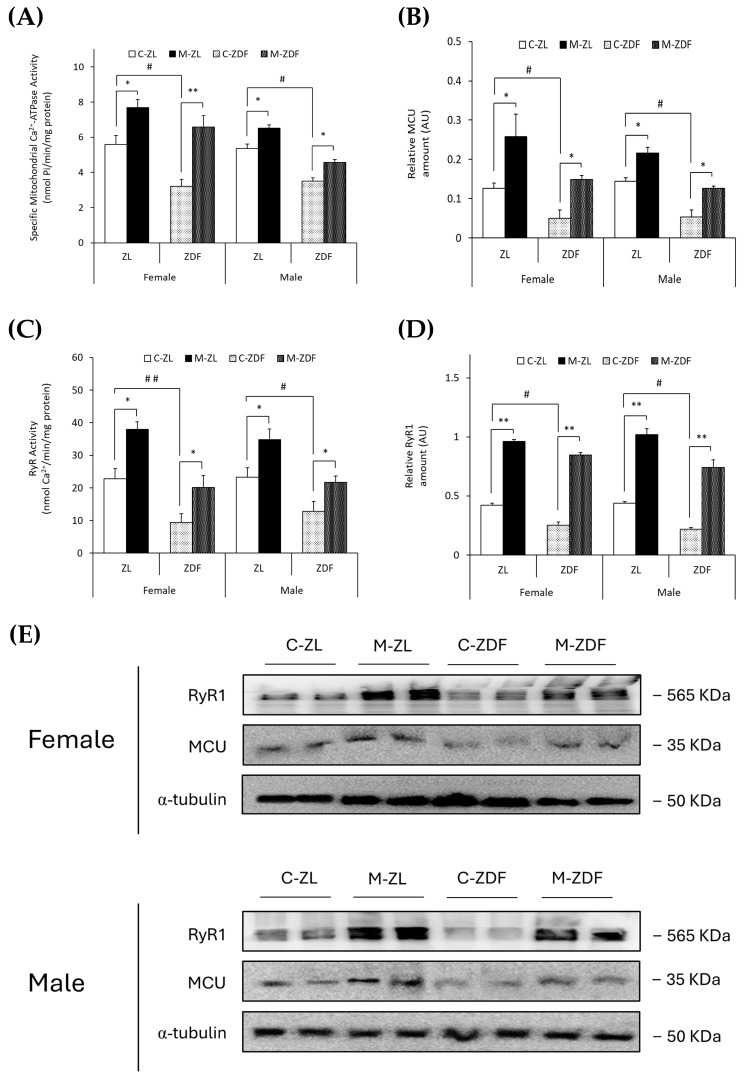
Melatonin’s effects on the main VL organellar calcium transporters from female and male Zücker lean (ZL) and Zücker diabetic fatty (ZDF) rats. (**A**) Specific mitochondrial Ca^2+^-ATPase activity (nmol Pi/min/mg protein). (**B**) Densitometry quantification of mitochondrial calcium uniporter (MCU) expression. (**C**) Endoplasmic reticulum ryanodine receptor (RyR) activity (nmol Ca^2+^/min/mg protein). (**D**) Densitometry quantification of RyR1 expression. (**E**) Representative blots of MCU and RyR1 protein expression. Results are expressed as mean ± S.E.M. of three independent experiments in duplicate. One-way ANOVA and Tukey’s post-test were performed for statistical analysis (* *p* < 0.05 and ** *p* < 0.01 melatonin vs. control rats; # *p* < 0.05 and ## *p* < 0.01 C-ZDF vs. C-ZL rats).

The specific mitochondrial Ca^2+^-ATPase activity (nmol Pi/min/mg protein) and MCU expression (AU) were observed to be diminished in obese animals of both sexes (female, 3.22 ± 0.39 nmol Pi/min/mg protein; 0.05 ± 0.02 AU; male, 3.50 ± 0.20 nmol Pi/min/mg protein; 0.06 ± 0.02 AU, respectively) compared to lean control littermates (female, 5.60 ± 0.51 nmol Pi/min/mg protein; 0.13 ± 0.01 AU; male, 5.37 ± 0.24 nmol Pi/min/mg protein; 0.14 ± 0.01 AU; *p* < 0.05), and melatonin treatment completely recovered the mitochondrial Ca^2+^-ATPase activity (female, *p* < 0.01; male, *p* < 0.05) and MCU expression (female and male, *p* < 0.05), even increasing them in both sexes of ZL treated rats (female, 7.69 ± 0.45 nmol Pi/min/mg protein; 0.26 ± 0.06 AU; male, 6.51 ± 0.20 nmol Pi/min/mg protein; 0.22 ± 0.03 AU; *p* < 0.05; for mitochondrial Ca^2+^-ATPase activity and MCU expression, respectively; Figure 2A,B). After protein isolation, total protein amount and α-Tubulin were not observed to change significantly between any groups (Figure 2E).

Moreover, as shown in Figure 2C,D, RyR activity (nmol Ca^2+^/min/mg protein) and expression (AU) were found to be diminished in both sexes of obese diabetic rats (female, 9.47 ± 2.60 nmol Ca^2+^/min/mg protein; 0.25 ± 0.03 AU; male, 12.83 ± 3.01 nmol Ca^2+^/min/mg protein; 0.22 ± 0.01 AU, respectively) in contrast with control ZL female (RyR activity, 22.81 ± 3.09 nmol Ca^2+^/min/mg protein; *p* < 0.01; RyR1 expression, 0.42 ± 0.01 AU; *p* < 0.05) and male rats (RyR activity, 23.32 ± 2.88 nmol Ca^2+^/min/mg protein; RyR1 expression, 0.44 ± 0.02 AU; *p* < 0.05). Melatonin treatment increased RyR activity in both sexes and phenotypes of rats (female M-ZDF, 20.15 ± 3.59 nmol Ca^2+^/min/mg protein; M-ZL, 37.93 ± 2.32 nmol Ca^2+^/min/mg protein; male M-ZDF, 21.72 ± 1.96 nmol Ca^2+^/min/mg protein; M-ZL, 34.82 ± 3.26 nmol Ca^2+^/min/mg protein; *p* < 0.05; Figure 2C) and RyR1 expression (female M-ZDF, 0.85 ± 0.02 AU; M-ZL, 0.96 ± 0.03 AU; male M-ZDF, 0.74 ± 0.06 AU; M-ZL, 1.02 ± 0.05 AU; *p* < 0.01; Figure 2D). This modulatory effect of melatonin on calcium homeostasis, increasing the MCU and RyR1 expression in VL, is also evident in the blots shown in Figure 2E.

### 3.2. Effects of Melatonin on VL ER Stress Response and UPR Pathway Activation

**Figure 3:**
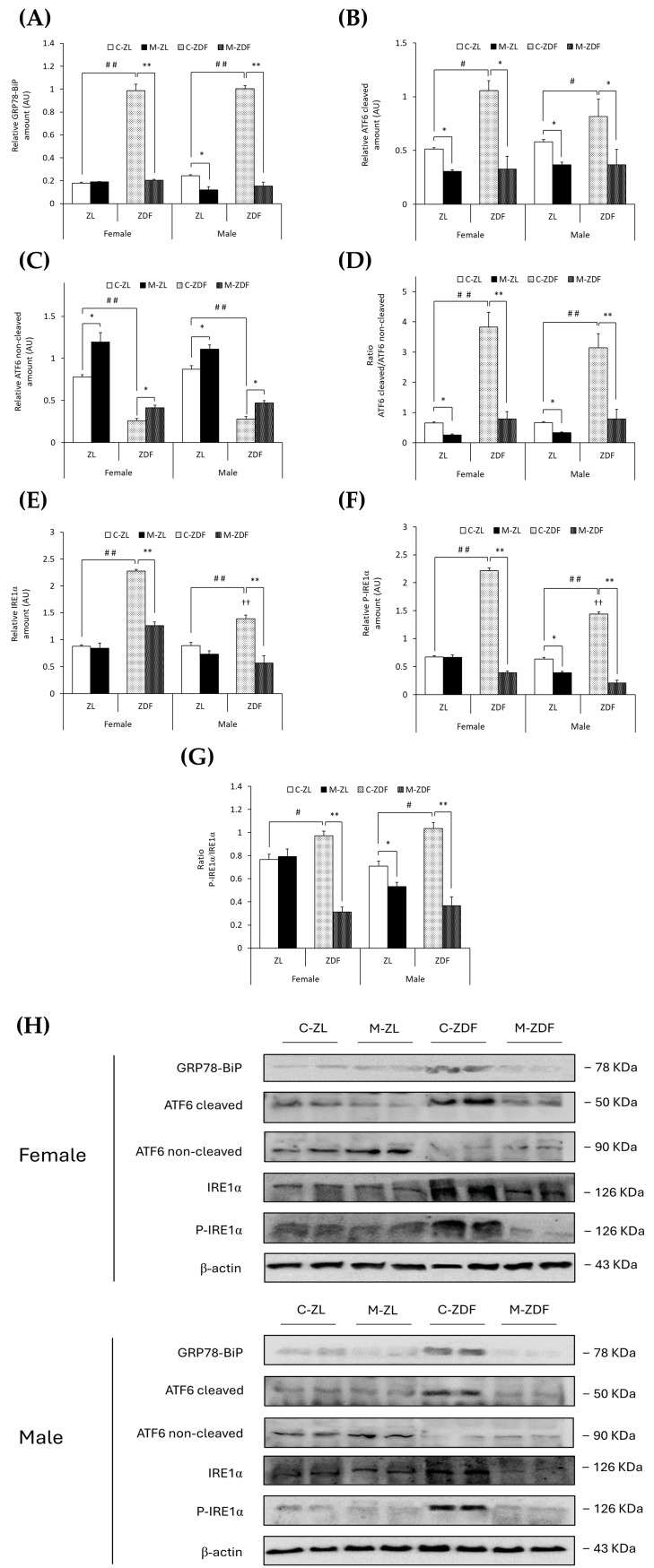
Melatonin’s effects on the endoplasmic reticulum (ER) stress: ATF6 and IRE1α unfolded protein response (UPR) pathway activation in the VL from female and male Zücker lean (ZL) and Zücker diabetic fatty (ZDF) rats. (**A**) Densitometry quantification of GRP78-BiP expression. (**B**) Densitometry quantification of cleaved ATF6 (activated free form) amount. (**C**) Densitometry quantification of non-cleaved ATF6 (non-activated membrane form) amount. (**D**) Ratio of cleaved/non-cleaved ATF6 amount. (**E**) Densitometry quantification of IRE1α expression. (**F**) Densitometry quantification of P-IRE1α expression. (**G**) Ratio of P-IRE1α/IRE1α (activated form (phosphorylated)/total) expression. (**H**) Representative blots of GRP78-BiP, ATF6, IRE1α, and P-IRE1α protein expression. Results are expressed as mean ± S.E.M. of three independent experiments in duplicate. One-way ANOVA and Tukey’s post-test were performed for statistical analysis (* *p* < 0.05 and ** *p* < 0.01 melatonin vs. control rats; # *p* < 0.05 and ## *p* < 0.01 C-ZDF vs. C-ZL rats; †† *p* < 0.01 female C-ZDF vs. male C-ZDF).

GRP78-BiP protein expression was about five times higher in the C–ZDF group compared to the C–ZL group in both sexes of rats (*p* < 0.01), and melatonin significantly decreased GRP78 expression in both sexes of ZDF rats, reaching lean counterpart expression levels (*p* < 0.01), and also in male ZL rats (0.12 ± 0.03 AU; *p* < 0.05; Figure 3A). In addition, in Figure 3B,C, obese diabetic animals had higher levels of cleaved ATF6, and thus lower levels of non-cleaved ATF6, in both sexes of ZDF rats (female, 1.06 ± 0.09 AU; 0.26 ± 0.03 AU; male, 0.82 ± 0.16 AU; 0.28 ± 0.03 AU, respectively) compared to their lean control animals (cleaved ATF6 female, 0.51 ± 0.01 AU; male, 0.58 ± 0.02 AU; *p* < 0.05; non-cleaved ATF6 female, 0.78 ± 0.03 AU; male, 0.87 ± 0.04 AU; *p* < 0.01). Therefore, the ratio of cleaved/non-cleaved ATF6 was significantly increased in both sexes of ZDF rats compared to their respective C-ZL groups (*p* < 0.01; Figure 3D). After melatonin treatment, in both sexes and phenotypes, the ATF6 cleavage was reduced (female M-ZDF, 0.33 ± 0.12 AU; M-ZL, 0.31 ± 0.01 AU; male M-ZDF, 0.37 ± 0.14 AU; M-ZL, 0.37 ± 0.03 AU; *p* < 0.05; Figure 3B), and non-cleaved ATF6 levels increased (female M-ZDF, 0.41 ± 0.03 AU; M-ZL, 1.20 ± 0.11 AU; male M-ZDF, 0.47 ± 0.03 AU; M-ZL, 1.11 ± 0.05 AU; *p* < 0.05; Figure 3C), also decreasing the ratio of cleaved/non-cleaved ATF6 in both sexes of ZDF rats (*p* < 0.01) and ZL rats (*p* < 0.05), as shown in Figure 3D.

Notably, comparing with ZL rats, both sexes of ZDF rats exhibited enhanced expression levels of both IRE1α (female C-ZDF, 2.28 ± 0.03 AU vs. C-ZL, 0.88 ± 0.03 AU; male C-ZDF, 1.39 ± 0.07 AU vs. C-ZL, 0.90 ± 0.05 AU; *p* < 0.01; Figure 3E) and P-IRE1α (female C-ZDF, 2.21 ± 0.05 AU vs. C-ZL, 0.68 ± 0.02 AU; male: C-ZDF, 1.44 ± 0.04 AU vs. C-ZL, 0.64 ± 0.03 AU; *p* < 0.01; Figure 3F), as well as higher levels of IRE1α and P-IRE1α in obese diabetic females than in males (*p* < 0.01). Consequently, the ratio of P-IRE1α/IRE1α was higher in both sexes of ZDF rats compared to their respective lean groups (*p* < 0.05; Figure 3G). Melatonin treatment diminished both IRE1α and P-IRE1α expression in both sexes of ZDF rats (female, 1.26 ± 0.07 AU and 0.39 ± 0.03 AU; male, 0.57 ± 0.13 AU and 0.21 ± 0.04 AU; *p* < 0.01; Figure 3E,F, respectively), and the P-IRE1α/IRE1α ratio levels were also lower in obese diabetic animals (*p* < 0.01; Figure 3G). Furthermore, as shown in Figure 3F,G, melatonin decreased P-IRE1α expression and the ratio of P-IRE1α/IRE1α in lean male ZL rats (0.39 ± 0.02 AU and 0.53 ± 0.04, respectively; *p* < 0.05).

**Figure 4:**
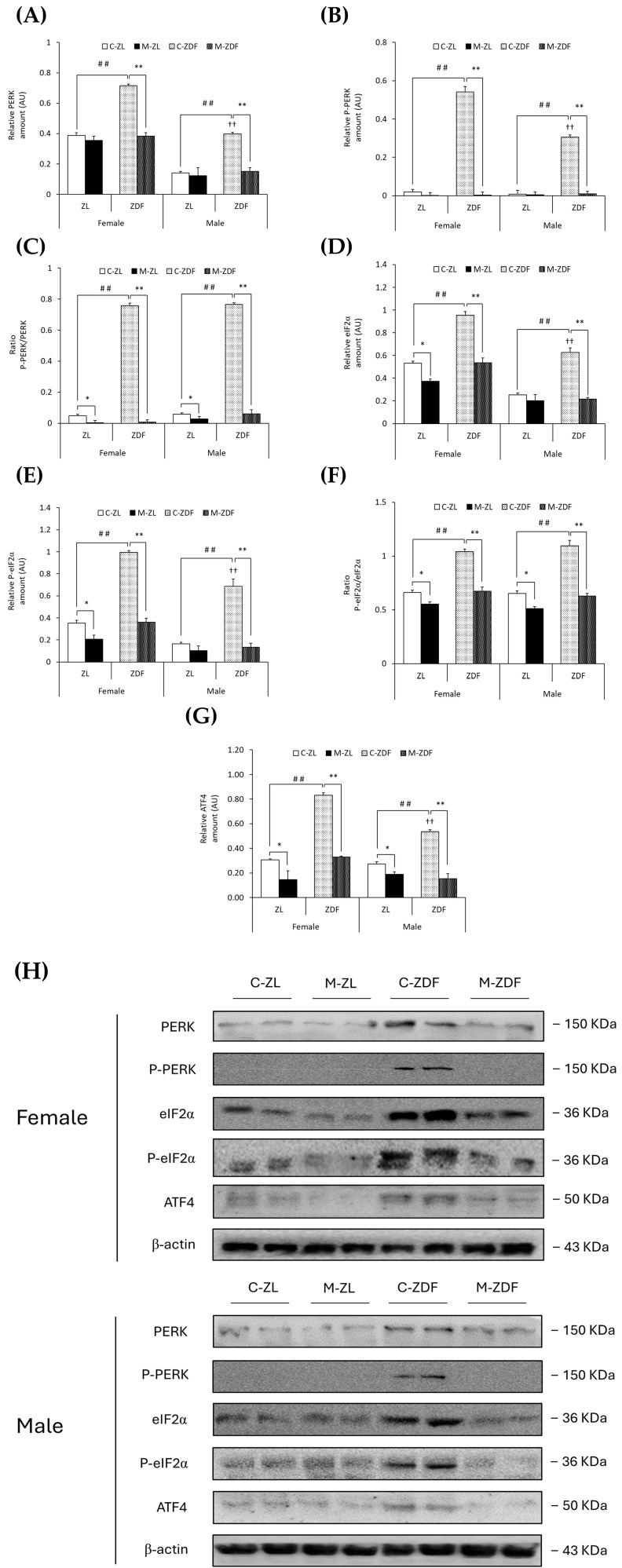
Melatonin’s effects on endoplasmic reticulum (ER) stress: PERK unfolded protein response (UPR) pathway activation in the VL of female and male Zücker lean (ZL) and Zücker diabetic fatty (ZDF) rats. (**A**) Densitometry quantification of PERK expression. (**B**) Densitometry quantification of P-PERK expression. (**C**) Ratio of P-PERK/PERK (activated form (phosphorylated)/total) expression. (**D**) Densitometry quantification of eIF2α expression. (**E**) Densitometry quantification of P-eIF2α expression. (**F**) Ratio of P-eIF2α/eIF2α (activated form (phosphorylated)/total) expression. (**G**) Densitometry quantification of ATF4 expression. (**H**) Representative blots of PERK, P-PERK, eIF2α, P-eIF2α, and ATF4 protein expression. Results are expressed as mean ± S.E.M. of three independent experiments in duplicate. One-way ANOVA and Tukey’s post-test were performed for statistical analysis (* *p* < 0.05 and ** *p* < 0.01 melatonin vs. control rats; ## *p* < 0.01 C-ZDF vs. C-ZL rats; †† *p* < 0.01 female C-ZDF vs. male C-ZDF).

Moreover, PERK expression was upregulated in both sexes of ZDF rats (female C-ZDF, 0.72 ± 0.01 AU vs. C-ZL, 0.39 ± 0.02 AU; male C-ZDF, 0.40 ± 0.01 AU vs. C-ZL, 0.14 ± 0.01 AU; *p* < 0.01; Figure 4A), and the P-PERK levels in the same rats were also found to be increased (female C-ZDF, 0.54 ± 0.03 AU vs. C-ZL, 0.02 ± 0.01 AU; male C-ZDF, 0.30 ± 0.01 AU vs. C-ZL, 0.01 ± 0.01 AU; *p* < 0.01; Figure 4B), as well as the P-PERK/PERK ratio when compared to ZL control rats (*p* < 0.01; Figure 4C). PERK and P-PERK expression was higher in female ZDF rats than in males (*p* < 0.01). As a consequence of melatonin treatment, the protein expression levels of PERK and P-PERK were lower in the VL of female (0.38 ± 0.02 AU and 0.01 ± 0.02 AU, respectively; *p* < 0.01) and male ZDF rats (0.15 ± 0.02 AU and 0.01 ± 0.01 AU, respectively; *p* < 0.01), as showed in Figure 4A,B, respectively, also reducing the ratio P-PERK/PERK in the same rats (*p* < 0.01) and even decreasing this ratio in both sexes of ZL rats (female, 0.01 ± 0.01; male, 0.03 ± 0.02; *p* < 0.05; Figure 4C).

As expected, obese diabetic ZDF rats of both sexes presented higher expression levels of eIF2α (female, 0.95 ± 0.03 AU; male, 0.63 ± 0.04 AU) and P-eIF2α (female, 0.99 ± 0.02 AU; male, 0.69 ± 0.07 AU) compared to their respective lean control animals (eIF2α female, 0.53 ± 0.02 AU; male, 0.25 ± 0.02 AU; P-eIF2α female, 0.35 ± 0.03 AU; male, 0.16 ± 0.01 AU; *p* < 0.01; Figure 4D,E, respectively). The ratio of P-eIF2α/eIF2α was also higher in both sexes of ZDF rats than in ZL rats (*p* < 0.01; Figure 4F). The protein expression of eIF2α and P-eIF2α in female ZDF rats was found to be increased when compared to male ZDF rats (*p* < 0.01). After melatonin treatment, eIF2α and P-eIF2α expression, and therefore the ratio of P-eIF2α/eIF2α, were decreased in the VL of female and male obese diabetic animals (*p* < 0.01), reaching healthy lean rats’ expression levels, and even in the VL of female ZL rats (eIF2α, 0.37 ± 0.02 AU; P-eIF2α, 0.21 ± 0.04 AU; *p* < 0.05), as shown in Figure 4D,E, respectively. Furthermore, melatonin reduced the P-eIF2α/eIF2α ratio in both sexes of ZL rats (*p* < 0.05; Figure 4F).

Moreover, both sexes of ZDF rats showed upregulation of ATF4 protein compared to lean rats (female C-ZDF, 0.83 ± 0.02 AU vs. C-ZL, 0.31 ± 0.01 AU; male C-ZDF, 0.53 ± 0.02 AU vs. C-ZL, 0.27 ± 0.02 AU; *p* < 0.01), with greater ATF4 expression in ZDF females than in males (*p* < 0.01; Figure 4G). Melatonin treatment, as illustrated in Figure 4G, diminished ATF4 expression in both sexes of ZDF rats (female, 0.33 ± 0.01 AU; male, 0.15 ± 0.04 AU; *p* < 0.01), also lowering the ATF4 protein levels in lean melatonin-treated animals (female, 0.15 ± 0.07 AU; male, 0.19 ± 0.02 AU; *p* < 0.05). These melatonin effects on VL ER stress-response-activating GRP78-BiP expression, and the ATF6-, IRE1α-, and PERK-mediated UPR pathway in the VL of both sexes of ZDF rats, are also illustrated in the blots shown in Figure 3H and Figure 4H.

### 3.3. Effects of Melatonin on VL ER Stress-Mediated Apoptosis

**Figure 5:**
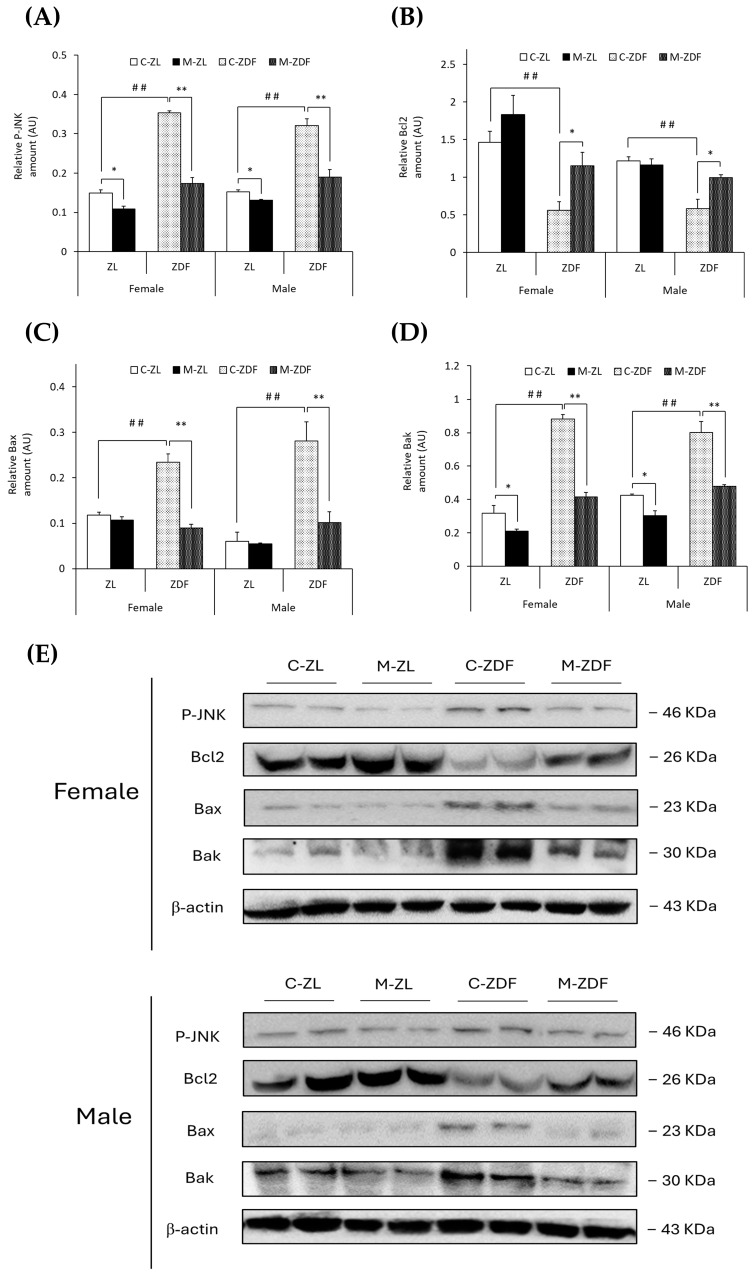
Melatonin’s effects on the apoptosis-regulation pathway in the VL of female and male Zücker lean (ZL) and Zücker diabetic fatty (ZDF) rats. (**A**) Densitometry quantification of P-JNK expression. (**B**) Densitometry quantification of Bcl2 expression. (**C**) Densitometry quantification of Bax expression. (**D**) Densitometry quantification of Bak expression. (**E**) Representative blots of P-JNK, Bcl2, Bax, and Bak protein expression. Results are expressed as mean ± S.E.M. of three independent experiments in duplicate. One-way ANOVA and Tukey’s post-test were performed for statistical analysis (* *p* < 0.05 and ** *p* < 0.01 melatonin vs. control rats; ## *p* < 0.01 C-ZDF vs. C-ZL rats).

It was observed that the levels of phosphorylated forms of JNK protein were higher in both sexes of ZDF rats than their respective lean control rats (female C-ZDF, 0.35 ± 0.01 AU vs. C-ZL, 0.15 ± 0.01 AU; male C-ZDF, 0.32 ± 0.02 AU vs. C-ZL, 0.15 ± 0.01 AU; *p* < 0.01; Figure 5A). Nevertheless, melatonin was found to decrease P-JNK expression in both sexes of ZDF (female, 0.17 ± 0.01 AU; male, 0.19 ± 0.02 AU; *p* < 0.01) and ZL rats (female, 0.11 ± 0.01 AU; male, 0.13 ± 0.01 AU; *p* < 0.05; Figure 5A). Consequently, Bcl2 expression was diminished in both sexes of ZDF rats (female, 0.56 ± 0.12 AU; male, 0.58 ± 0.12 AU) compared to their respective lean control rats (female, 1.46 ± 0.14 AU; male, 1.22 ± 0.05 AU; *p* < 0.01), and melatonin treatment recovered their Bcl2 expression, almost reaching lean counterparts’ levels (*p* < 0.05; Figure 5B). However, Bax protein levels were increased in obese diabetic animals from both sexes compared to their respective lean littermates (female C-ZDF, 0.23 ± 0.02 AU vs. C-ZL, 0.12 ± 0.01 AU; male C-ZDF, 0.28 ± 0.04 AU vs. C-ZL, 0.06 ± 0.02 AU; *p* < 0.01; Figure 5C). After melatonin treatment, Bax expression was recovered in both sexes of ZDF rats (*p* < 0.01; Figure 5C). In addition, Bak expression was found to be enhanced in both sexes of ZDF rats (female, 0.88 ± 0.03 AU; male, 0.80 ± 0.07 AU) compared to ZL control rats (female, 0.32 ± 0.05 AU; male, 0.42 ± 0.01 AU; *p* < 0.01; Figure 5D). Melatonin completely reversed the effects of diabesity on Bak expression in both sexes of ZDF rats (*p* < 0.01) and further reduced Bak protein levels in both sexes of ZL rats (female, 0.21 ± 0.01 AU; male, 0.30 ± 0.03 AU; *p* < 0.05), as shown in Figure 5D.

**Figure 6:**
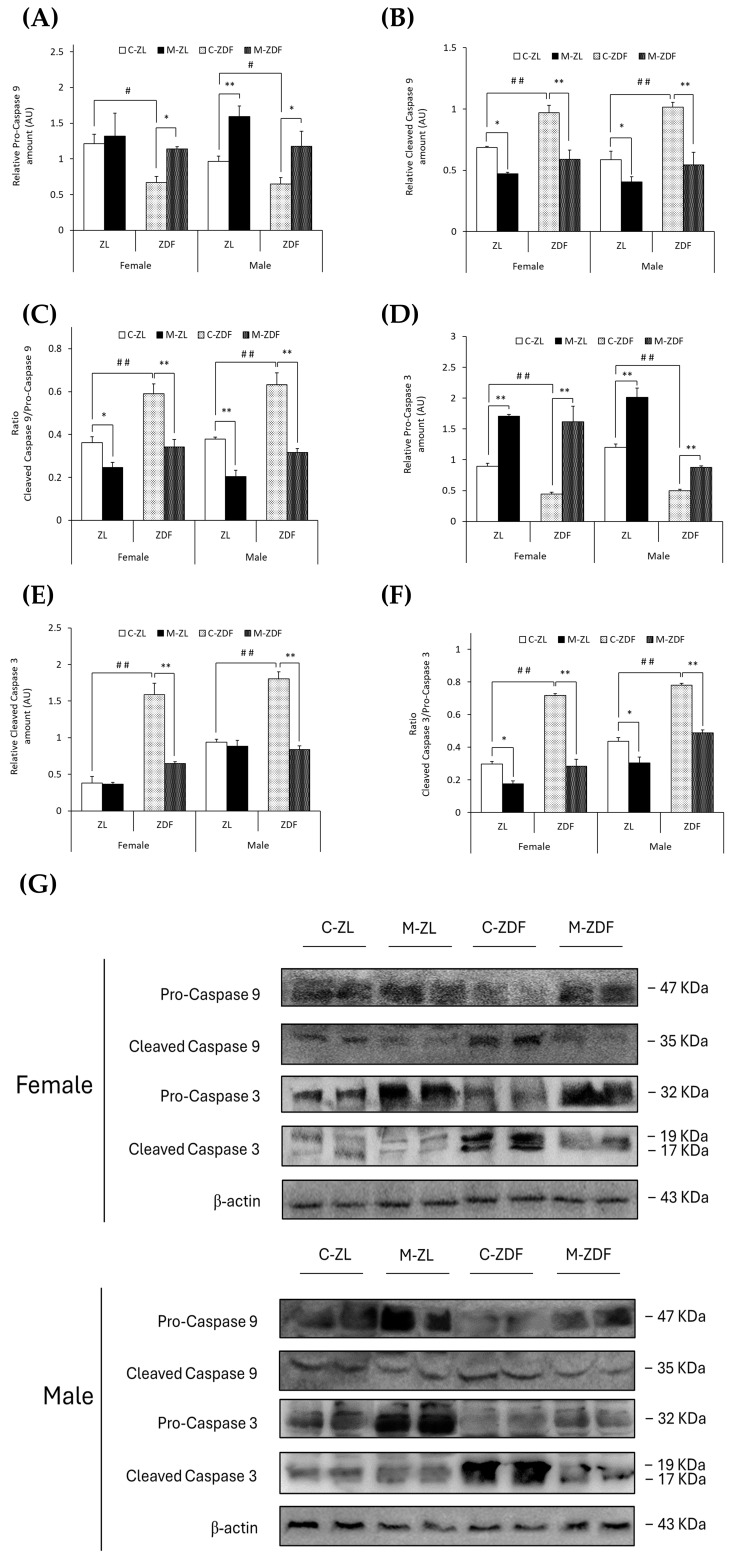
Melatonin’s effects on the activation of the apoptosis execution pathway by cleavage of caspases in the VL of female and male Zücker lean (ZL) and Zücker diabetic fatty (ZDF) rats. (**A**) Densitometry quantification of pro-caspase 9 (non-activated form) amount. (**B**) Densitometry quantification of cleaved caspase 9 (activated free form) amount. (**C**) Ratio of cleaved caspase 9/pro-caspase 9 amount. (**D**) Densitometry quantification of pro-caspase 3 (non-activated form) amount. (**E**) Densitometry quantification of cleaved caspase 3 (activated free form) amount. (**F**) Ratio of cleaved caspase 3/pro-caspase 3 amount. (**G**) Representative blots of caspase 9 and caspase 3 protein expression. Results are expressed as mean ± S.E.M. of three independent experiments in duplicate. One-way ANOVA and Tukey’s post-test were performed for statistical analysis (* *p* < 0.05 and ** *p* < 0.01 melatonin vs. control rats; # *p* < 0.05 and ## *p* < 0.01 C-ZDF vs. C-ZL rats).

In both sexes of ZDF rats, (non-cleaved) pro-caspase 9 levels were decreased (female C-ZDF, 0.67 ± 0.08 AU vs. C-ZL, 1.21 ±0.14 AU; male C-ZDF, 0.65 ± 0.09 AU vs. C-ZL, 0.97 ± 0.07 AU; *p* < 0.05; Figure 6A), and thus, cleaved caspase 9 levels were increased (female C-ZDF, 0.97 ± 0.06 AU vs. C-ZL, 0.69 ± 0.01 AU; male C-ZDF, 1.02 ± 0.04 AU vs. C-ZL, 0.59 ± 0.07 AU; *p* < 0.01; Figure 6B). Melatonin treatment completely recovered pro-caspase 9 and cleaved caspase 9 levels in both sexes of ZDF rats (pro-caspase 9, *p* < 0.05; cleaved caspase 9, *p* < 0.01), increased pro-caspase 9 levels in male ZL rats (1.59 ± 0.15 AU; *p* < 0.01; Figure 6A), and decreased cleaved caspase 9 levels in both sexes of ZL rats (female, 0.47 ± 0.01 AU; male, 0.41 ± 0.04 AU; *p* < 0.05; Figure 6B). Therefore, the ratio of cleaved caspase 9/pro-caspase 9 was found to be increased in both sexes of ZDF rats (female, 0.59 ± 0.04; male, 0.63 ± 0.06) compared to their lean littermates (female, 0.36 ± 0.03; male, 0.38 ± 0.01; *p* < 0.01), and melatonin restored the ratio levels in obese diabetic animals, reaching lean counterparts’ levels (*p* < 0.01), further diminishing the cleaved caspase 9/pro-caspase 9 ratio in ZL female (*p* < 0.05) and male rats (*p* < 0.01), as showed in Figure 6C.

In parallel, (non-cleaved) pro-caspase 3 levels were lower in both sexes of ZDF rats (female, 0.44 ± 0.03 AU; male, 0.50 ± 0.02 AU) than in ZL rats (female, 0.89 ± 0.05 AU; male, 1.20 ±0.05 AU; *p* < 0.01; Figure 6D). However, cleaved caspase 3 and the ratio cleaved caspase 3/pro-caspase 3 were found to be higher in both sexes of ZDF rats (female, 1.59 ± 0.15 AU and 0.72 ± 0.01; male, 1.80 ± 0.09 AU and 0.78 ± 0.01) than in their respective lean control animals (cleaved caspase 3 female, 0.38 ± 0.09 AU; male, 0.94 ± 0.04 AU; ratio cleaved caspase 3/pro-caspase 3 female, 0.30 ± 0.01; male, 0.44 ± 0.02; *p* < 0.01; Figure 6E,F, respectively). After melatonin treatment, pro-caspase 3 levels were increased in both sexes and phenotypes of animals (female M-ZDF, 1.62 ± 0.26 AU; M-ZL, 1.71 ± 0.03 AU; male M-ZDF, 0.88 ± 0.03 AU; M-ZL, 2.01 ± 0.15 AU; *p* < 0.01; Figure 6D). Moreover, melatonin inhibited the cleavage of caspase 3 in obese diabetic animals, reducing cleaved caspase 3 levels in the VL of both sexes of ZDF rats (female, 0.64 ± 0.03 AU; male, 0.84 ± 0.05 AU; *p* < 0.01; Figure 6E). Consequently, melatonin completely restored the cleaved caspase 3/pro-caspase 3 ratio in both sexes of ZDF rats (*p* < 0.01), as well as reducing this ratio’s levels in both sexes of ZL rats (*p* < 0.05), as shown in Figure 6F. The effects of melatonin downregulating apoptosis in the VL of female and male ZDF rats can also be observed in the blots in Figure 5E and Figure 6G.

### 3.4. Effects of Melatonin on VL Melatonin Receptors

**Figure 7:**
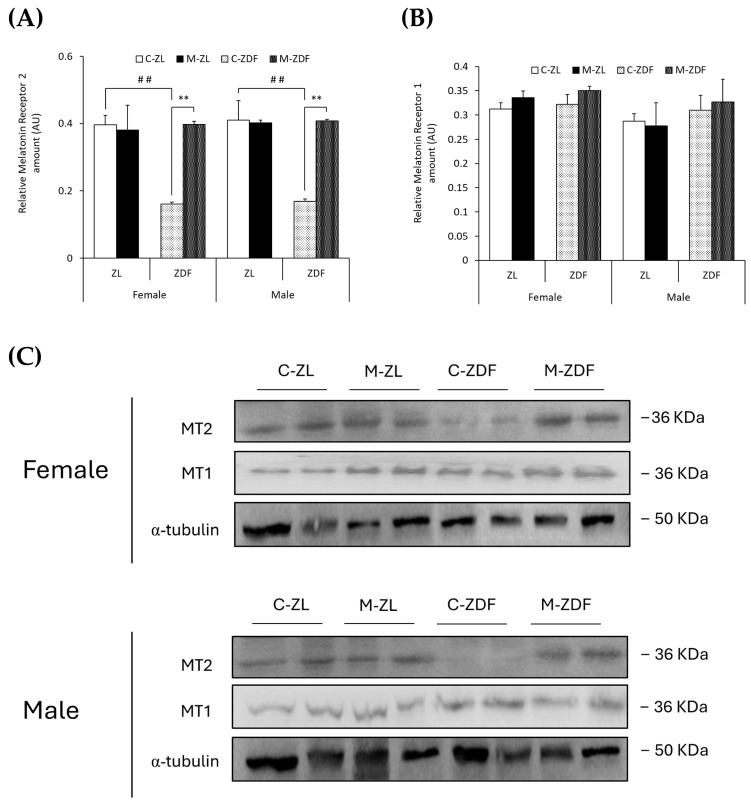
Melatonin’s effects on melatonin receptor expression in the VL of female and male Zücker lean (ZL) and Zücker diabetic fatty (ZDF) rats. (**A**) Densitometry quantification of melatonin receptor 2 (MT2) amount. (**B**) Densitometry quantification of melatonin receptor 1 (MT1) amount. (**C**) Representative blots of MT2 and MT1 protein expression. Results are expressed as mean ± S.E.M. of three independent experiments in duplicate. One-way ANOVA and Tukey’s post-test were performed for statistical analysis (** *p* < 0.01 melatonin vs. control rats; ## *p* < 0.01 C-ZDF vs. C-ZL rats).

In both sexes of ZDF rats, MT2 levels were lower compared to lean counterparts (female C-ZDF, 0.16 ± 0.01 AU vs. C-ZL, 0.39 ± 0.03 AU; male C-ZDF, 0.17 ± 0.01 AU vs. C-ZL, 0.41 ± 0.06 AU; *p* < 0.01; Figure 7A), and melatonin treatment increased MT2 expression, counteracting the effects of diabesity and reaching lean rats’ levels (female, 0.40 ± 0.01 AU; male, 0.41 ± 0.01 AU; *p* < 0.01; Figure 7A). As shown in Figure 7B, MT1 expression was not significantly different in both sexes and phenotypes of ZDF rats, and melatonin also did not modify MT1 expression. The effects of melatonin on MT2 and MT1 expression in the VL of female and male ZDF rats are also illustrated in Figure 7C.

## 4. Discussion

The results from the present work confirmed that melatonin plays an important role in calcium homeostasis in the SKM of obese diabetic rodents, as shown in a previous study by our group [18]. More precisely, we demonstrated that chronic melatonin administration modulates intraorganellar calcium concentrations, specifically in mitochondria and ER, two main organelles in cellular calcium balance. This restored calcium homeostasis observed in the present study after melatonin treatment was associated with organellar function and stress response restoration accompanied by reduced cytosolic and mitochondrial oxidative stress status in the SKM [18,19]. There was also a decrease in both mitochondria and ER stress responses followed by the subsequent apoptosis inhibition, as shown in the present and previous studies by our group. Based on these data and previous findings, we suggest that melatonin’s therapeutic benefits in diabesity, recovering the SKM structure, mass, and function [12,19], could be attributed, at least in part, to the relief of SKM ER stress-mediated apoptosis. Moreover, in the present study, no significant changes were observed in the behavior of treated ZDF rats at a chronic high pharmacological melatonin dose of 10 mg/kg/day. Additionally, feeding and drinking patterns were stable (Supplementary Table S1), as reported in previous studies in the same rat strain at the same melatonin dose and time [19,35]. Rats were not sleeping all the time, locomotor activity did not change [41], and habits were normal with no significant variations. These data are in agreement with the results of other groups in which high-dose melatonin treatment did not alter locomotor activity or behavior, produce a sedative effect, or result in changes in sleeping patterns in rats [42,43,44]. However, there are inconsistencies regarding the effects of high-dose melatonin treatment in rodents in terms of circadian rhythm. More specific and deep research is needed to better understand the beneficial effects of melatonin as a circadian regulator in the same present study conditions as well as the safety of the treatment.

Mitochondrial calcium homeostasis regulates cell metabolism, mitochondrial function, and energy production, being essential in mitochondrial stress-mediated apoptosis [45]. When SKM mitochondria are exposed to high oxidative stress levels, as occurs in metabolic diseases such as obesity and T2DM, the mitochondrial membrane integrity is compromised, leading to calcium release into the cytosol [46]. This decreases intramitochondrial calcium levels, as shown in the present study, in which intramitochondrial calcium levels in both sexes of obese diabetic ZDF rats were found to be lower in the VL muscle, explaining the increased ROS production and mitochondrial dysfunction in the VL [19], and in other insulin-sensitive tissues such as the liver [47] and adipose tissue [48] from the same rat strain as our preceding study. To better understand these processes in diabesity and the protective role of melatonin in SKM, further studies on mitochondrial membrane integrity are needed. Because of the toxicity of high cytosolic calcium concentrations, this ion is removed from the cytosol by the uptake of ER calcium pumps and stored in this organelle [49] increasing intra-ER calcium levels as showed in the VL from both sexes of obese diabetic animals. Higher intraorganellar calcium levels were also reported in obese [50,51] and diabetic rodents [52]. Here, we demonstrate for the first time that melatonin increases in mitochondria and decreases in ER the intraorganellar calcium levels in the VL of both sexes of ZDF rats, showing its modulatory role on intraorganellar calcium homeostasis and reversing diabesity effects. Further studies in intraorganellar melatonin levels and the influence of this indolamine on cellular calcium dynamic and transporter activity will help in the understanding of the organellar modulatory effects of melatonin in SKM under obese diabetic conditions. Elevated mitochondrial calcium levels promote ATP synthesis and oxidative phosphorylation [45], aligning with our earlier results showing the beneficial effects of melatonin in mitochondrial functionality in the VL from obese diabetic ZDF rats [19].

Intraorganellar calcium homeostasis is regulated by transporters, mainly the specific mitochondrial Ca^2+^-ATPase and the MCU in mitochondria as well as the SERCA pump and RyR channel in the ER [6,7]. A recent study from our team showed that both sexes of ZDF rats presented lower protein levels and activity of the SERCA pump in the VL muscle [35]. Consistent with these results, here we show that the activity of another Ca^2+^-ATPase pump in mitochondria was also reduced, as was the expression of the MCU channel in the VL of both sexes of ZDF rats. Decreased MCU expression levels have been related to SKM dysfunction, impacting the development and progression of diabesity [53]. Moreover, RyR expression and activity were observed to be decreased in obese diabetic animals, these data being consistent with those found in diabetic mice [52] and obese rats [54] in which modifications in RyR structure were found, impacting channel functionality. A previous study in VL from the same rat strain showed that melatonin improves SERCA activity and expression [35], and consistent with this, the present results show that melatonin recovers mitochondrial Ca^2+^-ATPase and RyR activity and increases MCU and RyR1 protein levels, bringing to light the key role of melatonin in regulating organellar function via calcium signaling and preventing organellar stress-mediated cell death [14]. The current study has potential limitations, as it is important to emphasize that calcium regulation is a dynamic process, so further in vitro studies on human SKM models concerning intracellular calcium exchanges between organelles, specifically by ER–mitochondria interactions, and fine calcium dynamics regulation would improve understanding of melatonin’s role in SKM calcium homeostasis and ER stress in diabesity, bringing melatonin closer to current SKM clinical therapies under obese diabetic conditions.

ER stress is increased in SKM under obese diabetic conditions, contributing to the development and progression of SKM dysfunction [55]. ER stress markers such as GRP78-BiP, ATF6, P-IRE1α, P-PERK, and P-eIF2α were found to be elevated in several in vitro myoblast models of human obesity [56,57] as well as in the SKM from both sexes of obese and diabetic rodents [58,59] and obese and/or diabetic women [60]. The present data show that these markers are increased in the VL of both sexes of obese diabetic ZDF rats, demonstrating the close relationship between the diabesity condition and ER stress. However, melatonin treatment inhibits ER stress by reducing the activation (by cleavage or phosphorylation) of the main UPR response initiators (ATF6, IRE1α, and PERK) and thereby, down-regulating UPR markers, reversing diabesity’s effects. Coherent with the data obtained, in a diabetic myoblast in vitro model, melatonin reduced GRP78-BiP, P-PERK, and P-IRE1α levels [61]. Furthermore, melatonin inhibited ER stress in cardiac muscle, reversing the effects of diabetic cardiomyopathy in rodents [62]. In other insulin-sensitive tissues, melatonin also reduced ER stress by downregulating GRP78-BiP, P-PERK, P-IRE1α, ATF6, and ATF4 expression, as shown by a previous study in an in vitro model of a fatty liver [63] and in the kidneys of the same strain of obese diabetic rat [16] in which UPR activation led to apoptosis.

UPR pathway activation is a protective mechanism, which, when prolonged or excessive, can trigger apoptotic mechanisms to remove damaged SKM cells, as occurs in metabolic disorders such as diabesity [8]. The ER stress-mediated apoptosis pathway begins with the activation of the JNK protein by phosphorylation [9]. A previous study in a myoblast in vitro model of human obesity demonstrated that P-JNK expression was higher under this condition, upregulating ER stress and apoptotic signals [56]. Furthermore, several studies in both sexes of obese diabetic rodents also showed that ER stress in the SKM induces P-JNK activation with consequent higher pro-apoptotic Bax and lower anti-apoptotic Bcl2 protein expression [59,64,65]. This pro-apoptotic environment promotes the activation of SKM apoptosis by increasing the cleavage of caspases 9 and 3 in both sexes of obese diabetic rodents [59,64,65]. Consistent with these results, the present data show that the VL from both sexes of obese diabetic rats have higher levels of pro-apoptotic signals, exhibiting increased P-JNK, Bax, and Bak expression and lower levels of anti-apoptotic Bcl2 protein. Moreover, caspase 9 and 3 cleavage are shown to be up-regulated, decreasing both inactive pro-caspase forms and enhancing the apoptosis in the VL from both sexes of ZDF rats. Melatonin, conversely, increases Bcl2 levels, reducing P-JNK and pro-apoptotic proteins Bax and Bak expression and also lowering caspase activation, fully reversing the effects of diabesity in the VL from both sexes of ZDF rats. Therefore, our data show that melatonin inhibits the SKM ER stress-mediated apoptosis, which may explain the protective role of melatonin in diabesity observed in our previous study, preventing muscle mass and function loss [19]. In agreement with the presented data, in a diabetic myoblast in vitro model, melatonin reduced P-JNK protein, preventing myoblast dysfunction [61]. In diabetic mice, melatonin also increases Bcl2 expression and decreases Bax, caspase 9, and caspase 3 levels in cardiac muscle, reducing apoptosis [62,66]. The parallel effects of melatonin were found in injured muscles [67,68], which undergo inflammatory processes similar to those occurring in diabesity [69]. Furthermore, in an earlier study performed by our group using the kidney from the same rat strain, melatonin reduced ER stress-mediated apoptosis signaling, downregulating P-JNK, Bax, and cleaved caspase 3 and augmenting Bcl2 protein expression, preserving renal function and structure [16].

An increasing number of studies are bringing to light the close relationship between pathologies, such as diabetes and obesity conditions, with sleep disorders and circadian rhythm disturbances through what is currently known as circadian syndrome [70]. In line with these studies, and melatonin being the master regulator of circadian rhythms, results from the present study show that MT2 receptor expression, but not MT1, is decreased in the VL of both sexes of obese diabetic ZDF rats, suggesting that obesity and diabetes are highly related to circadian regulation by the MT2 receptor. Moreover, several studies associate this receptor with obesity and diabetes, illustrating that polymorphic variants of the MT2 gene are involved in melatonin’s effects on these conditions [71,72]. In the present study, we also show that melatonin increases MT2 expression in the VL from ZDF rats in accordance with the previously mentioned relationship between the effects of melatonin and the MT2 receptor. Regarding melatonin’s effects on ER stress, various mechanisms have been proposed to be involved in the direct (by melatonin’s interaction with UPR initiators (PERK, IRE1α, and/or ATF6) and/or by melatonin’s transcriptional regulation of UPR genes) or indirect (by melatonin’s binding to membrane MT1/MT2 receptors and/or decreasing ROS stress) influence of melatonin on UPR pathway regulation [32]. In previous studies on diabetic cardiomyopathy, MT2, but not MT1, was found to be upregulated after melatonin treatment, which reduced ROS and ER stress and improved cardiac function, in accordance with the data from the present study [73,74]. Furthermore, melatonin was found to relieve oxidative stress and ER stress-induced apoptosis in diabetic cardiomyopathy through SIRT1, AMPK, and PGC1α activation [75]. In previous studies from our group in the same rat strain, we also observed an increase in SIRT1 expression, AMPK activation through phosphorylation, P-AMPK/AMPK ratio, and PGC1α upregulation after melatonin treatment [19,35]. Therefore, our data suggest that melatonin binds to MT2, activating SIRT1, AMPK, and PGC1α and downregulating the UPR pathway and ER stress-mediated apoptosis activation. However, in the present study, we only measured the expression of membrane melatonin receptors without considering the activation of the cascade and whether the MT2 receptor and/or other melatonin receptors are required for the effects on melatonin to be reported. Further studies in knockout in vivo and/or knockdown in vitro models using siRNAs or CRISPR/CAS9 technologies are needed to elucidate the mechanisms that underly melatonin’s effects on SKM ER stress relief and calcium transport within cells.

## 5. Conclusions

Taking together our current and previous experimental results, they suggest that melatonin could emerge as a potential novel therapeutic agent for preventing muscle mass loss and atrophy and improving muscle function in diabetic and obese individuals by reducing cellular oxidative stress, which restores organellar function and therefore organellar calcium homeostasis, reducing ER stress-mediated apoptosis activation. Further studies on intracellular calcium dynamic and homeostasis regulation; calcium transport; and communication between organelles through specific cellular structures such as mitochondria-associated membranes (MAMs), which are essential in ER–mitochondria interactions, as well as mechanistic studies on the melatonin receptor involved in these processes with knockdown or knockout models, are needed for a better understanding of melatonin’s effects on calcium homeostasis and organellar stress. In addition, further validation of the effects of melatonin and other melatonergic agents on human SKM using in vitro models will help to better understand melatonin’s effects on SKM health and also to further elucidate the mechanism that governs melatonin’s effects through membrane melatonin receptors such as MT1 and/or MT2. In addition, to translate these promising findings into viable clinical therapies, we estimate that the effective therapeutic dose ranges from 140 to 160 mg per day, administered orally, for an average obese adult weighing about 100–120 kg. Based on human equivalent dose estimations [76], the optimal human dose for these melatonin effects would correspond to 1–1.5 mg/kg/day. More clinical research is needed with high melatonin doses to better elucidate the safety and efficacy of this indolamine due to the lack of toxicological studies performed by the scientific community. Clinical effects of melatonin in humans are very scattered and inconclusive as well as controversial, depending on the melatonin dose and time used. In several studies, no significant side effects of melatonin supplementation were reported even at high doses [77]; nevertheless, further robust randomized clinical trials and chronopharmacological studies as well as more studies on melatonin’s side effects in circadian rhythm regulation at this high pharmacological dose are essential to find out the effective dose and appropriate time of the day for the treatment.

## Data Availability

All data supporting the findings of this study are available within the paper and its Supplementary Information. Data generated are available from the corresponding author upon request.

## Supplementary Materials

The following supporting information can be downloaded at: https://www.mdpi.com/article/10.3390/antiox14010016/s1, Supplementary Table S1: Effects of melatonin treatment on water and total food intake (ml/day/rat) and vastus lateralis (VL) weight (g) in both ZL and Zücker diabetic fatty (ZDF) groups of both sexes, Supplementary Table S2: List of antibodies used for Western blot analysis.
