# Supplementary material for: Melatonin Ameliorates Organellar Calcium Homeostasis, Improving Endoplasmic Reticulum Stress-Mediated Apoptosis in the Vastus Lateralis Muscle of Both Sexes of Obese Diabetic Rats
Source: Antioxidants (Basel). 2024 Dec 26;14(1):16. doi: 10.3390/antiox14010016 (PMC11762543; doi:10.3390/antiox14010016)
Supplement: Supplementary file 1 [file antioxidants-14-00016-s001.zip › antioxidants-3324869-supplementary.pdf]

Supplementary Table S1. Effects of melatonin treatment on water and total food intake (ml/day/rat) and vastus lateralis (VL) weight (g) in both ZL and Zücker diabetic fatty (ZDF) groups of both sex female and male groups: control and melatonin-treated. Results were expressed as the mean and S.E.M., respectively (n = 8). One-way ANOVA followed by Tukey's post- test, (** *P* < 0.01 M-ZDF vs. C-ZDF; ## *P* < 0.01 C-ZDF vs C-ZL rats).

Sex	Phenotypes	Water intake (ml/day/rat)	Total Food intake (g/wk/rat)	VL weight (g)
Female	C-ZL	22.85 ± 0.50	88.85 ± 4.06	1.73 ± 0.32
	M-ZL	21.27 ± 0.63	85.04 ± 2.53	1.98 ± 0.68
	C-ZDF	97.08 ± 0.48 ##	197.62 ± 6.88 ##	1.39 ± 0.52
	M-ZDF	86.20 ± 0. 35 **	186.75 ± 9.86	1.81 ± 0.47
Male	C-ZL	21.75 ± 0.90	84.56 ± 6.58	2.76 ± 0.56
	M-ZL	21.29 ± 1.61	90.47 ± 7.24	2.96 ± 0.45
	C-ZDF	96.01 ± 0.67 ##	187.31 ± 4.50 ##	2.16 ± 0.11
	M-ZDF	83.26 ± 1.98 **	191.15 ± 3.84	2.51 ± 0.60

Supplementary Table S2. List of antibodies used for Western-Blot

ANTIGEN	PRODUCT CODE	COMPANY
RyR1	MA3-925	Thermofisher Scientific
CCDC109A/MCU	sc-515930	Santa Cruz Biotechnology
Caspase 3	sc-7148	Santa Cruz Biotechnology
Caspase 9	sc-133109	Santa Cruz Biotechnology
GRP78/BiP	G9043	Sigma-Aldrich
ATF6	sc-166659	Santa Cruz Biotechnology
IRE1α	SAB2500366	Sigma-Aldrich
P-IRE1α	AP0878	ABclonal Technology
PERK	P0074	Sigma-Aldrich
P-PERK	OASG05793	Aviva Systems Biology
eIF2α	AV41041	Sigma-Aldrich
P-eIF2α	SAB4504388	Sigma-Aldrich
ATF4	WH0000468M1	Sigma-Aldrich
P-JNK	07-175	Sigma-Aldrich
Bcl2	sc-7382	Santa Cruz Biotechnology
Bax	sc-7480	Santa Cruz Biotechnology
Bak	sc-517390	Santa Cruz Biotechnology
MEL-1A-R (MT1)	sc-13186	Santa Cruz Biotechnology
MEL-1B-R (MT2)	sc-13177	Santa Cruz Biotechnology
β-actin	sc-81178	Santa Cruz Biotechnology
α-tubulin	sc-5286	Santa Cruz Biotechnology
Donkey anti-Rabbit IgG (H+L), HRP	A16035	Thermofisher Scientific
Donkey anti-Mouse IgG (H+L), HRP	MBS674947	MyBiosource
Rabbit anti-Goat IgG (H+L), HRP	A5420	Sigma-Aldrich
